# Zsyntax: A Formal Language for Molecular Biology with Projected Applications in Text Mining and Biological Prediction

**DOI:** 10.1371/journal.pone.0009511

**Published:** 2010-03-03

**Authors:** Giovanni Boniolo, Marcello D'Agostino, Pier Paolo Di Fiore

**Affiliations:** 1 IFOM, Istituto FIRC di Oncologia Molecolare, Milano, Italy; 2 Dipartimento di Medicina, Chirurgia ed Odontoiatria, Università di Milano, Milano, Italy; 3 Dipartimento di Scienze Umane, Università di Ferrara, Ferrara, Italy; 4 Istituto Europeo di Oncologia, Milano, Italy; Center for Genomic Regulation, Spain

## Abstract

We propose a formal language that allows for transposing biological information precisely and rigorously into machine-readable information. This language, which we call Zsyntax (where Z stands for the Greek word ζωή, life), is grounded on a particular type of non-classical logic, and it can be used to write algorithms and computer programs. We present it as a first step towards a comprehensive formal language for molecular biology in which any biological process can be written and analyzed as a sort of logical “deduction”. Moreover, we illustrate the potential value of this language, both in the field of text mining and in that of biological prediction.

## Introduction

It is often claimed that biology needs to be formalized (see for instance the Special issue of *Science*, Mathematics in Biology, of Feb 6^th^ 2004 available at http://www.sciencemag.org/content/vol303/issue5659/index.dtl). In principle, there are many advantages that might be drawn from the implementation of a formal biological language, since formalization ensures non-ambiguity and a degree of precision that cannot be achieved by ordinary language. Indeed, there are numerous excellent examples of the application of mathematics to describe biological systems: take, for instance, the theory of graphs and, in particular, the progress made in the field of scale-free networks [1], [2], [3], [4], or the wide-spread use of the theory of differential equations to describe biological kinetics and dynamics, as any text-book of mathematical biology illustrates [5], [6], [7]. However, each of these applications is limited to the particular system it aims to describe. That is, fragments of mathematical knowledge are applied in function of the given biological situations to be analyzed.

The modeling of biochemical systems has also been addressed drawing on formal methods from computer science, by exploiting the analogy between biochemical reactions and computational processes. For example, intensive research has been carried out on extensions and adaptations of the π-calculus, a formalism originally developed for the specification of concurrent processes [8], [9], [10], [11], [12], [13] that can be used to model biochemical networks as mobile communication systems. Other groups have focused on developing software environments by means of a rule-based syntax that can be interpreted in terms of several reaction models, making use of techniques from (classical) temporal logic to formalize their properties and query the models [14], [15]. Moreover, important effort has been devoted to treat the well-known phenomenon of *combinatorial explosion*, i.e., the fact that the number of distinct states of protein complexes grows exponentially with the number of binding domains and interaction surfaces present in proteins, by introducing *macrostates*, i.e., quantitative indicators of cumulative properties of the system such as levels of occupancy or degrees of phosphorylation [16] or introducing approximation techniques, such as the layer-based approach [17].

These efforts have greatly improved our ability of modeling biochemical reactions by means of rigorous mathematical tools, leading to formalisms that are amenable to computer implementation. On the other hand, the formal and mathematical techniques involved, although biologically meaningful, may – in some cases – prove too difficult to grasp (and to implement) for the working biologist. For example, while arguing in favor of the Kappa-calculus, an extension of the π-calculus, Fontana admits that “the reduction of concepts from concurrency to biological practice is neither simple to implement nor easy for biologists to grasp. It deals with unfamiliar concepts, whose clarification took a long time even within their domain of origin” [9]. While we fully recognize the significant advances made in all these research areas, we argue – in this paper – for a *logical* approach to biochemical processes, by exploiting the analogy between such processes and *logical deductions*. We recognize that such an endeavor might meet with the same difficulties encountered by other formalizations, in terms of acceptance and usage by working biologists. For this reason we have attempted to construct and to propose our formalism in the most biologist-centered way. Since our main objective is to attract the attention of the working biologist, the present exposition aims at providing an informal account of the main ideas underlying the project, while a more detailed formal account, to the benefit of the logician and of the computer scientist, will be provided in a subsequent work.

In proposing our approach, we acknowledge the interesting previous efforts to develop a logical language for biology, based on *classical logic* [18], [19], [20]. We feel, however, that classical logic is unsuited to develop a heuristically useful language for biology, in particular for molecular biology. This latter field conceals a latent *non-classical logic* underlying theoretical and experimental reasoning that can be brought to light by focusing on the analogy between biochemical processes and logical deductions. From this point of view, logic is not just an auxiliary tool for analyzing biological models based on some external formalism, but becomes the core of a research program in which biological processes are the intended semantical interpretation of a non-classical logical system. The goal of our present quest has been exactly to draw out such logic and make it explicit. To this purpose, we have developed a basic formal language for molecular biology, which we call Zsyntax (where Z stands for the Greek word ζωή, life). This language is grounded on a particular type of non-classical logic and should be seen as the first step towards the full specification of a real-scale formal language that can be used to write algorithms and computer programs. Once fully developed, such a real-scale language will allow us to exploit the expertise that has been accumulated in the last few decades in the field of automated theorem-proving, in order to develop, in the future, new and more efficient predictive tools than those currently available. In this paper, we restrict ourselves to the basic notions, indicating a “roadmap for the future” that is different from that suggested by other formal approaches.

In addition to biological prediction, we believe that the power of a logical approach can be applied to other fields of great relevance to biology, in particular to text mining. The explosive growth in data availability has confronted text mining with major hurdles in the retrieval, extraction, and compaction of relevant information [21]. There is, therefore, great interest in the development of efficient computable tools. By and large, text-mining strategies are focused on processing natural language, which is the means by which most molecular biology papers are written. We propose a different starting basis for text mining: if biological processes could be universally transposed into a formal, unambiguous logical language, text mining would benefit from the added precision, non-ambiguity and amenability to computer processing that the latter would provide, compared to natural language.

In this paper, we introduce Zsyntax and show some important features it possesses, in particular:

- It provides a mathematically rigorous representation of molecular biology processes. The formalism we employ, which can account also for reaction stoichiometry, represents aggregates of molecules as logical formulae. These formulae are assembled into chains, in accordance with rigorous logical rules (i.e. through logical inferences), to represent chains of biological reactions, so that the latter are treated as logical deductions.
- It can be used to focus in a concise way on the core of most molecular biology papers, namely on the description of biological processes. This may offer a good basis to text miners for the construction of tools to retrieve, extract and compact biological information.
- It is heuristic (in the sense that it fosters discovery), as it allows us to structure prediction problems as problems of “filling the gaps” in an incomplete deduction, i.e. when we know the end point of a biological process, but need to work out some missing data in the start. In logical literature this is known as “abduction”, a process that has been investigated thoroughly from the computational viewpoint.
- It is computer-implementable, which means that it may allow researchers to capitalize on the growing body of research carried out in the field of automated deduction, which aims to create computer programs to demonstrate theorems.

## Results and Discussion

### Defining the Formal Language Zsyntax

Biological processes are usually described in terms of their participant molecules (simple ones, such as glucose, or more complex ones such as enzymes, or genes). If two types of molecules, A and B, are able to interact in some way, we denote the outcome of this interaction as A*B, where by the “star” sign * we indicate the operator between A and B that we call *Z-interaction*. In this case we have a binary operation that is defined only for pairs of types of molecules A and B that interact. In general, the operation * is not associative, since it may happen that the reaction (A*B) *C is different from the reaction A* (B*C). For, although A interacts with B, and the resulting product A*B interacts with C, it may not be true that B interacts with C, and so neither B*C nor A* (B*C) exist. This is a common situation in enzymology and in gene regulation. For example, in the case of the Trp Operator of *E. coli*, the Trp-repressor does not bind to the Operator if it is not bound to Tryptophan. In other words, (Tryptophan*Trp-repressor) *Operator ≠ Tryptophan* (Trp-repressor*Operator), the latter being a condition that does not exist.

We can also describe an aggregate of *n* molecules of types A_1_, …, A_n_. In this case, the aggregate can be denoted by introducing the operator called the *Z-conjunction* and graphically indicated by the ampersand, &. In this case the aggregate becomes A_1_ & … & A_n_. It is important to note that molecules in an aggregate do not necessarily react. However, if A and B are types of interacting molecules, any aggregate of type A & B will yield a compound molecule of type A*B, under suitable bio-physical conditions, and given enough time. As before, we may regard “&” as a binary operation between types of molecules.

It should be noted that there is an important formal difference between the Z-conjunction, as herein defined, and the classical conjunction. While classically – where we are concerned with propositions – the conjunction between the proposition A and A itself has the same content as the proposition A, in Zsyntax – which deals with types of molecules, that is, with physical resources – the type A & A is by no means the same as the type A, since it refers to aggregates of two molecules of type A. Hence, Z-conjunction is not idempotent. Note that this property of the Z-conjunction allows Zsyntax to take into account the stoichiometry of a reaction, since it permits to consider the exact number of molecules of a certain type needed in that reaction. This is connected with the fact that an aggregate A_1_& ^…^ &A_n_ does not represent a set, but a *multiset* of formulae (sometimes called a “bag”). This means that each member within the set can occur several times. For example, the multiset containing two occurrences of A, three occurrences of B and one occurrence of C is represented as A&A&B&B&B&C, not as A&B&C. In this way we can take into account the number of times a type of molecule occurs, in order to formalize reaction stoichiometry correctly. Finally note that, unlike *, & is fully associative and, therefore, does not require parentheses, so both A&(B&C) and (A&B)&C can be written as A&B&C.

The third and final operator that we use in Zsyntax is the *Z-conditional*, denoted by the sign → (that we call “arrow”). To grasp its meaning let us consider an initial aggregate of molecules A&C and a final aggregate B. In this case, we say that all aggregates of type C are also of type A → B (A “arrow” B), in that they map any aggregate of type A into an aggregate of type B. That is, there is a transition, or a path, from A to B if there is C allowing it (this is the reason why we say that → is a Z-conditional).

At this point we can claim that Zsyntax consists of the set of formulae built out of the atomic, i.e. non-reducible or elementary, formulae by means of the operators &_,_ *_,_ → Note that while the Z-interaction is defined only between types of (single) molecules, the Z-conjunction and the Z-conditional are defined between arbitrary types, including types of aggregates of molecules.

Having defined the basics of the language, now it is necessary to introduce the notion of *validity*, which allows us to affirm that a given formula is a valid one. We claim that a formula A → B is valid if the empty aggregate (namely the aggregate consisting of zero molecules), denoted by 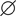, allows the path from A to B; that is, if 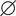
&A → B. An example may be helpful to clarify the underlying idea. Consider the case of the tumor suppressor TP53 [22]. We know that TP53 can bind the *MDM2* gene, to activate its transcription, the ultimate consequence being that the MDM2 protein is produced in the cell [23]. In this case there is an intermediate product, that is, MDM2*TP53. Then, by definition of the Z-conditional, the “empty aggregate”, 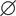, is of type *MDM2*&TP53 → MDM2; since from any aggregate of type 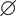 & *MDM2*&TP53  =  *MDM2*&TP53 we can arrive at some aggregate of type MDM2. Under these circumstances we say that the formula *MDM2*&TP53 → MDM2 is valid.

Thanks to the notion of ‘valid formula’ just given, we can claim that Zsyntax is constructed from two basic kinds of valid formulae:

#### 1. Empirically valid formulae (EVF)

These represent reactions, and their validity depends only on empirical information acquired in the laboratory. So the processes that EVFs represent have been empirically corroborated. Examples of basic EVFs include (i) two molecules that interact (e.g. *MDM2*&TP53 → MDM2*TP53); (ii) two molecules that interact to deliver biochemical products (e.g. D-Glucose-6-phosphate*Glucose-6-phosphate isomerase → D-Fructose-6-phosphate), or to deliver the products of gene expression (e.g. MDM2*TP53 → MDM2). The validity of these formulae is *content-dependent*: this means that if the molecules in a valid formula are changed, the formula will not necessarily remain valid. Note that empirically valid formulae are to be considered as the non-logical axioms of a molecular biology theory.

#### 2. Logically valid formulae (LVF)

These formulae give the rules of logic that govern the transition from one EVF to the next. Only LVFs are *formally* valid, and their validity depends only on the definitions of the logical operators used within Zsyntax, regardless of the molecules involved. In this sense, their validity is *content-independent*, so it is preserved under uniform substitution of data-types with arbitrary variables. A LVF in our language is the equivalent of a *tautology* in classical logic, that is, a formula which is derivable from the “empty set of assumptions”. LVFs are to be thought of as the logical axioms of a molecular biology theory. Some basic LVFs can be presented in terms of “inference rules” that regulate the application of the operators and the transition from one formula to the next when describing a biological process, as discussed in the next section.

At this point we have, on the one hand, biological processes and, on the other, a formal language by means of which we can precisely and rigorously encode information concerning biological reactions in a set of EVFs. We can also move from one EVF to another, by means of inferences (i.e., deductions) justified on the grounds of LVFs.

A logical language of this kind allows us to write biological processes in a format that is precise, rigorous and comprehensible. Moreover, and more importantly, the symbols &, * and → *obey general laws that are formally analogous to logical laws*, as will be shown in the next section. This allows us to represent *biological* processes as *logical* processes. In logic, we start with a premise and, by deduction, we reach a conclusion. Analogously in Zsyntax we start with reactants and, by using empirically and logically valid rules, we reach the product of the reaction, i.e. we have deductions where the premises represent the reactants (the initial aggregate) and the conclusion represents the (aggregate of the) products.

As already noticed when commenting on the definition of the Z-conjunction operator, the underlying logic governing the behavior of the above operators is not classical, but belongs to a family of non-classical resource-aware systems, called “substructural logics”, which have received a good deal of attention in the field of computational logic [24], [25]. Therefore, the intensive research that has been carried out in this area – as far as the development of efficient automated deduction algorithms is concerned – can be exploited to provide new methods of information processing for biological applications.

### Biological Reactions as Logical Inferences

In the previous section we briefly introduced the analogy between biological reactions and logical deductions. This section serves to explain the parallels between the two in greater detail. In particular, we can say that an initial aggregate (IA) A_1_& …&A_n_ *implies* a final aggregate B, if the latter is *derivable* from the former by means of an inferential process (i.e. a deduction) whose steps are allowed by EVFs and by LVFs. We can write this as:
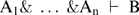
where “⊢” (that can be read ‘implies’) is what we call the *derivability relation* which indicates that B is derivable from A_1_ & …& A_n_, that is, that there is a path from A_1_ & … & A_n_ to B. Importantly, this derivability relation satisfies the two fundamental properties of reflexivity and transitivity, which are widely recognized as sufficient conditions for the relation ⊢ to represent a logical system [26]. Note that the derivability relation is clearly transitive, in that from A_1_ & …& A_n_ ⊢ B_1_ & …& B_m_ and B_1_ & …& B_m_ ⊢ C_1_ & …& C_r_, it follows that A_1_ & …& An ⊢ C_1_ & …& C_r_ (for all A_1_, …, A_n_, B_1_, …, B_m_, C_1_, …, C_r_). Moreover, it can be assumed that ⊢ is reflexive, so that A_1_ & …& A_n_ ⊢ A_1_ & …& A_n_ (for all A_1_, …, A_n_). Furthermore, it also satisfies the analogue of the so-called “deduction theorem” for the Z-conditional operator (→), which is valid in many logical systems, that is, given any aggregates A, B, C: 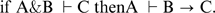



The definitions of the logical operators → and & justify a set of basic LVFs that can thought of as “logical axioms”. These can be presented in the form of intuitive rules that explain how these operators can be eliminated or introduced in a deductive inference. Such rules can be considered as rules of a non-standard system of natural deduction [27]:

1. **Elimination of the Z-conditional (that we indicate by →** ***E*** **).** If A→B can be derived from C and A can be derived from D, then B can be derived from C&D.
2. **Introduction of the Z-conditional (that we indicate by →I).** If B can be derived from C&A, then A → B can be derived from C alone. In the logical jargon one says that the “assumption” A is “discharged” by the application of such a rule, which in our context means that the availability of an aggregate of type A is incorporated as a (sufficient) condition in the antecedent of A → B. Hence, the derivability of (an aggregate of type) A → B no longer depends on the availability of (an aggregate of type) A.
3. **Elimination of Z-conjunction (that we indicate by &** ***E*** **).** If the Z-conjunction of A and B (A&B) can be derived from C, then both A and B individually can be derived from C.
4. **Introduction of Z-conjunction (that we indicate by &** ***I*** **).** If A can be derived from C, and B can be derived from D, then the conjunction of A and B can be derived from C&D.

There are no purely logical rules for the Z-interaction *. The reason is that * is not a purely logical operator, since its behavior depends on empirical information acquired in the laboratory. Therefore, * cannot be governed by *formal* rules, but rather, it can be introduced and eliminated only via EVFs of the form A&B→A*B (indicating that we obtain a compound from initially separate molecules) or A*B→C&D (indicating that we obtain two products from the division of an initial compound). Empirically valid formulas, however, can be fruitfully replaced by “empirically valid rules” whenever the context requires a more precise representation of additional information. See the next section for a brief discussion of this point.

A derivation, in this natural deduction system for biological reasoning, is simply a sequence of formulas that are either EVFs or result from the application of the above rules in order to logically represent an entire biological process. However, there is one important restriction that must be respected: any formula occurring in a line cannot be used more than once. This is because Zsyntax describes biological processes, or paths, in which reactants are “consumed”, or otherwise engaged, following a precise stoichiometry. Once they have been accounted for in a process or path, these particular molecules are no longer available, and if more are necessary, then new identical reactants must be introduced, or obtained again.

Combining in a correct way EVFs with the logical rules allows us to represent biological processes as deductions that establish *theorems* of the form A_1_& … &An ⊢ B. Below, in Table 1, we provide a comparison of the standard linguistic interpretation of the theorem A_1_& … &An ⊢ B with our non-standard biological interpretation.

**Table 1:** The linguistic and the biological interpretations of the theorems.

		LINGUISTIC CASE	BIOLOGICAL CASE (ZSYNTAX)
From	**PREMISES (hypotheses)**	**Conjunction of statements**	**Aggregate of molecules**
Through	Inferential process	Classical logical rules and non-logical axioms	Non-standard logical rules and EVFs
To	**CONCLUSION (thesis)**	**Statement**	**Aggregate of molecules**

An example of how this is achieved is illustrated in Table 2 and Table 3, where the reactions of the glycolytic pathway leading from D-Glucose to Fructose-1,6-bisphosphate are depicted in Zsyntax. In Table 2, the reactions are written in a strictly formal way, integrating EVFs with logical rules. The same pathway is presented in Table 3 in a simplified form that contains only the essential sequence of EVFs extracted from the detailed version. Table 2 and Table 3 also illustrate how simply Zsyntax can accommodate the fact that some of the reactants, in particular enzymes, are not “consumed” in the reaction; as a consequence, when these molecules are invoked in an EVF, they appear both in the reactants and in the metabolites.

**Table 2:** Theorem representing the reactions of the glycolytic pathway leading from D-Glucose to Fructose-1,6-bisphosphate: Glc&HK&GPI&PFK&ATP&ATP ⊢ F1,6P.

1. Glc & HK & GPI & PFK & ATP & ATP	IA
2. Glc & HK	From 1 by &*E*
3. GPI	From 1 by &*E*
4. PFK	From 1 by &*E*
5. ATP	From 1 by &*E*
6. ATP	From 1 by &*E*
7. Glc & HK → Glc*HK	EVF
8. Glc*HK	From 2,7 by →*E*
9. (Glc*HK) & ATP	From 5,8 by &*I*
10. (Glc*HK) & ATP → (Glc*HK) *ATP	EVF
11. (Glc*HK) *ATP	From 9,10 by →*E*
12. (Glc*HK) *ATP → G6P & HK & ADP	EVF
13. G6P & HK & ADP	From 11,12 by →*E*
14. G6P	From 13 by &*E*
15. HK	From 13 by &*E*
16. ADP	From 13 by &*E*
17. G6P & GPI	From 3,14 by &*I*
18. G6P & GPI → G6P*GPI	EVF
19. G6P*GPI	From 17,18 by →*E*
20. G6P*GPI → F6P & GPI	EVF
21. F6P & GPI	From 19,20 by →*E*
22. F6P	From 21 by &*E*
23. GPI	From 21 by &*E*
24. F6P & PFK	From 4,22 by &*I*
25. F6P & PFK → F6P*PFK	EVF
26. F6P*PFK	From 24,25 by →*E*
27. (F6P*PFK) & ATP	From 6,26 by &*I*
28. (F6P*PFK) & ATP → (F6P*PFK) *ATP	EVF
29. (F6P*PFK) *ATP	From 27,28 by →*E*
30. (F6P*PFK) *ATP → F1,6P & PFK & ADP	EVF
31. F1,6P & PFK & ADP	From 29,30 by →*E*
32. F1,6P	From 31 by &*E*
**33. (Glc & HK & GPI & PFK & ATP & ATP) → F1,6P**	From 1–32 by →*I*

The reactions of the pathway are illustrated in all their detail. In each line we write the conclusion of a rule application, together with its justification, without keeping track of the initial aggregates (IA) on which it may depend. All the lines in this example depend on the IA of line 1, except for the EVFs, which do not depend on any IA, and the final theorem reported on line 33. Here, the IA of line 1 is “discharged” by the application of rule →*I*, as indicated to the right of line 33 (From 1–32 by →*I*), with the consequence that the final theorem does not depend on any IA. Abbreviations: D-Glucose, Glc; D-Glucose-6-phosphate, G6PPP; Hexokinase [EC 2.7.1.1], HK; Glucose-6-phosphate isomerase [EC 5.3.1.9], GPI; 6-Phosphofructokinase [EC 2.7.1.11], PFK; Fructose-6-phosphate, F6PP; Fructose-1,6-bisphosphate, F1,6PP. Note that no formula is used more than once in the derivation process.

**Table 3:** Simplified version of the theorem.

**Theorem**
Glc&HK&GPI&PFK&ATP&ATP ⊢ F1,6P
**Demonstration**
1. Glc & HK → Glc*HK
2. (Glc*HK) & ATP → (Glc*HK) *ATP
3. (Glc*HK) *ATP → G6P & HK & ADP
4. G6P & GPI → G6P*GPI
5. G6P*GPI → F6P & GPI
6. F6P & PFK → F6P*PFK
7. (F6P*PFK) & ATP → (F6P*PFK) *ATP
8. (F6P*PFK) *ATP → F1,6P & PFK & ADP

In Zsyntax, deductions can be written in a simpler way than that presented in Table 2. Here, the emphasis is on the main steps of the inferential process, while inferential rules remain hidden. These rules must however be considered to be implicitly applied, in spite of the fact that they are not explicitly mentioned. Abbreviations are as in Table 2.

Additional examples are provided in Table 4, Table 5, Table 6 and Table 7, to illustrate more fully how Zsyntax can accurately depict stoichiometries, as well as complex biological interplays, such as regulatory or feed-forward loops.

**Table 4:** Theorem representing the regulatory loop involving *MDM2*, MDM2 and TP53 and leading to TP53 degradation: TP53& TP53& *MDM2&* U& P ⊢ d(TP53).

1. TP53 & TP53 & *MDM2* & U & P	IA
2. TP53	From 1 by &*E*
3. TP53	From 1 by &*E*
4. *MDM2*	From 1 by &*E*
5. U	From 1 by &*E*
6. P	From 1 by &*E*
7. TP53 & *MDM2*	From 2,4 by &*I*
8. TP53 & *MDM2* → TP53*MDM2	EVF
9. TP53*MDM2	From 7,8 by →*E*
10. TP53*MDM2 → MDM2	EVF
11. MDM2	From 9,10 by →*E*
12. MDM2 & TP53	From 3,11 by &*I*
13. MDM2 & TP53 → MDM2*TP53	EVF
14. MDM2*TP53	From 12,13 by →*E*
15. (MDM2*TP53) & U	From 5,14 by &*I*
16. (MDM2*TP53) & U → (MDM2*TP53) *U	EVF
17. (MDM2*TP53) *U	From 15,16 by →*E*
18. (MDM2*TP53) *U → MDM2 & (TP53*U)	EVF
19. MDM2 & (TP53*U)	From 17,18 by →*E*
20. TP53*U	From 19 by &*E*
21. (TP53*U) & P	From 6,20 by &*I*
22. (TP53*U) & P → (TP53*U) *P	EVF
23. (TP53*U) *P	From 21,22 by →*E*
24. (TP53*U) *P → d(TP53) & U & P	EVF
25. d(TP53) & U & P	From 23,24 by →*E*
26. d(TP53)	From 25 by &*E*
**27. (TP53 & TP53 & ** ***MDM2*** ** & U & P) → d(TP53)**	From 1–26 by →*I*

It is known that TP53, the well-known tumor suppressor [22] binds to the *MDM2* gene and activates its transcription, ultimately leading synthesis of the MDM2 protein [23], [28]. But if TP53 binds the protein MDM2, this latter acts as a ubiquitin ligase, leading to TP53 ubiquitination and ultimately to its proteasomal degradation [29], an event that we indicate by d(TP53). Thus, a complex regulatory loop exists involving TP53, the *MDM2* gene and the MDM2 protein. The reactions of this pathway are illustrated, in Zsyntax language, in the detailed form. In this form, the theorem is reported on line 27, the antecedent (IA, initial aggregate) is the multiset reported on line 1 and is “discharged” by the application of →***I***. Abbreviations: U, ubiquitin; P, proteasome. The reader can check that no formula (resource) is used more than once in the derivation process.

**Table 5:** Simplified version of the theorem representing the regulatory loop.

**Theorem**
TP53& TP53& *MDM2&* U& P ⊢ d(TP53)
**Demonstration**
1. TP53 & *MDM2* → TP53*MDM2
2. TP53 * *MDM2* → MDM2
3. MDM2 & TP53 → MDM2*TP53
4. (MDM2 *TP53) & U → (MDM2*TP53) *U
5. (MDM2*TP53) *U → MDM2 & (TP53*U)
6. (TP53*U) & P → (TP53*U) *P
7. (TP53*U) *P → d(TP53) & U & P

**Table 6:** Theorem representing the feed forward loop: *A & A & B & C &* RA& RA& RB ⊢ C.

1. *A* & *A* & *B* & *C* & RA & RA & RB	IA
2. *A*	From 1 by &*E*
3. *A*	From 1 by &*E*
4. *B*	From 1 by &*E*
5. *C*	From 1 by &*E*
6. RA	From 1 by &*E*
7. RA	From 1 by &*E*
8. RB	From 1 by &*E*
9. *A* &RA	From 2,6 by →*I*
10. *A* & RA → A*RA	EVF
11. A*RA	From 9,10 by →*E*
12. A*RA → A	EVF
13. A	From 11,12 by → *E*
14. A & RB	From 8,13 by &*I*
15. A & RB → A*RB	EVF
16. A*RB	From 14,15 by →*E*
17. (A*RB) & *B*	From 4,16 by &*I*
18. (A*RB) & *B* → (A*RB) *B	EVF
19. (A*RB) *B	From 17,18 by →*E*
20. (A*RB) *B → B	EVF
21. B	From 19,20 by →*E*
22. *A* & RA	From 3,7 by &*I*
23. *A* & RA → A*RA	EVF
24. A*RA	From 22,23 by →*E*
25. A*RA → A	EVF
26. A	From 24,25 by →*E*
27. A & B	From 21,26 by &*I*
28. A & B → A*B	EVF
29. A*B	From 27,28 by →*E*
30. (A*B) & *C*	From 5,29 by &*I*
31. (A*B) & *C* → (A*B) *C	EVF
32. (A * B) *C	From 30,31 by →*E*
33. (A * B) *C → C	EVF
34. C	From 32,33 by →*E*
**35. (** ***A*** ** & ** ***A*** ** & ** ***B*** ** & ** ***C*** ** & RA & RA & RB) → C**	From 1–34 by →*I*

A feed forward loop [30] is illustrated in the detailed form. In this form, the theorem is reported on line 35, the antecedent (IA, initial aggregate) is the multiset reported on line 1 and is “discharged” by the application of →***I***. The theorem illustrates the abstract case of a feed forward loop composed of three genes *A, B, C*, their encoded proteins (A, B, C), and two regulatory proteins RA and RB, such that (i) *A* is regulated by RA; (ii) *B* by RB and the protein A; (iii) *C* by the protein complex A*B. The reader can check that each formula (resource) is used at most once.

**Table 7:** Simplified version of the theorem representing the feed forward loop.

**Theorem**
*A & A & B & C &* RA& RA& RB ⊢ C
**Demonstration**
1. *A* & RA → A*RA
2. *A* * RA → A
3. A & RB → A*RB
4. (A*RB) & *B* → A*RB*B
5. (A*RB) *B → B
6. *A* & RA → A*RA
7. A*RA → A
8. A & B → A*B
9. (A*B) & *C* → (A*B) *C
10. (A*B) *C → C

When biological processes are described in this way, they can, to all intents and purposes, be treated as theorems, with a set of initial assumptions Γ and a conclusion B. We can therefore prove the validity of any biological “theorem” Γ ⊢ B by detailing the logical path leading from Γ to B, in much the same way as we prove the validity of a mathematical theorem. Of course, the examples we have provided are meant to illustrate the potential of Zsyntax, and they are, therefore, extremely simple. The real power of Zsyntax will be fully appreciable when it is applied to the design of a computer program that can deal with much more complex pathways and processes.

## Analysis

### Can Zsyntax Grasp the Complexity of Molecular Biology

It is not immediately intuitive that a language encompassing only three operators (&_,_ *_,_ →) can render the intricacy of molecular biology. While we do not claim, in principle, that all of molecular biology can be described through Zsyntax, our language is versatile enough to address several issues of biological complexity, as exemplified by the followings:

#### 1. Context dependence

Strictly speaking, this is not a topic directly dealing with biological complexity, but rather with the ambiguity regarding its representation. However, we find it useful to deal with it at first, since it provides the paradigm of how Zsyntax addresses the complexity issue by means of contextualization. For instance, Zsyntax can reclaim issues connected to synonymy (many names for the same molecule) and homonymy (many molecules for the same name). This is resolved by the fact that any Zsyntax theorem refers to a molecular context in which the ambiguity is dissolved. Also in the case in which the same molecule has different functions in different molecular or cellular contexts, Zsyntax can disambiguate the situation since any theorem refers to a specific molecular context. From its demonstration, therefore, we can infer the proximal function of that particular molecule. Of course the longer is the chain of reactions in the theorem the more we know also about the less proximal functions of the same molecule. Finally, as we will show below, by contextualizing the Zsyntax operators (point 4), or its derivability relation (point 5), or also its formulas (point 6), a number of relevant biological features can be represented and/or disambiguated.

#### 2. Post-translational modifications

This is a frequent occurrence in molecular biology, with significant impact on biological processes. Zsyntax can incorporate these instances. As an example, we will consider a situation already analyzed, that of the interaction between MDM2 and TP53. Phosphorylation of either one of the molecules can impair their interaction. For the sake of simplicity let us consider only the case in which TP53 is phosphorylated (see for instance [31]). This reaction can be written in Zsyntax as can be seen in Table 8.

**Table 8:** Theorem concerning the phosphorylation of TP53.

**Theorem**
TP53 & ATP*&* Kinase ⊢ TP53-P
**Demonstration**
1. Kinase & ATP → Kinase*ATP
2. (Kinase*ATP) & TP53 → (Kinase*ATP) *TP53
3. (Kinase*ATP) *TP53 → TP53-P & Kinase & ADP

Note that in this reaction, the actual kinase is unknown and it is therefore indicated with the general term “kinase”. In addition, it is not known the exact hierarchy of the binding of ATP and TP53 to the kinase (in other words, TP53 might bind before ATP, or vice versa). For the practical purpose of this demonstration, however, these gaps in knowledge are irrelevant. Note also that Zsyntax can accommodate an even higher level of resolution than what is depicted here. For instance, it is known that the phosphorylation event in question is on Ser18 of TP53. This can be expressed easily in Zsyntax, by further disambiguating the entity TP53-P in the theorem with the notation TP53-PS18.

At this point, it is rather self evident that, being TP53 and TP53-P two distinct entities, their interaction (or lack thereof) with MDM2 can be described by two separate expressions:

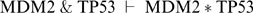

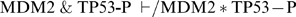

where by ⊢/ we mean that the antecedent (in this case, MDM2 & TP53-P) does not imply the consequent (in this case, MDM2 * TP53-P).

In a similar way, Zsyntax can describe situations in which post-translational modifications (e.g. phosphorylation) affect the activity of a protein (e.g. by inactivating an enzyme). Let us assume that the enzyme E catalyzes the reaction (where A and B are substrate and product, respectively):
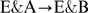



Let us also assume that the phosphorylated enzyme, E-P, is catalytically inactive. Again, being E and E-P two distinct entities, the reaction (or lack thereof) can be described by two separate expressions:

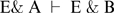

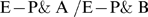

where, in ii), as above, the antecedent (E-P & A) does not imply the consequent (E-P & B).

#### 3. Allosteric configurations

Another instance in which disambiguation may be required concerns allosteric configurations. But even this case is grasped easily by Zsyntax. For, anytime we have an allosteric situation regarding *n* possible different configurations of the same protein, we have *n* different theorems – characterized by *n* different initial aggregates and by *n* different final aggregates – representing the *n* different situations. (It should be clear at this point that by appropriately disambiguating entities in the initial or final aggregates, virtually all instances can be reclaimed by Zsyntax. For instance, one could account for species (e.g., *H. sapiens*, *M. musculus*) by appropriately labeling the relevant molecules, for gene variation (SNPs), for splice variants and so on).

#### 4. Type of interaction

A frequently encountered problem concerns the type of methodology used experimentally to demonstrate an interaction between two molecules. This can be done in the wet lab through a series of methodologies, such as *in vitro* binding (with purified proteins or with one purified protein challenged with a cellular lysate), yeast two-hybrid, co-immunoprecipitation *in vivo*, FRET *in vivo*, and so on. The problem is relevant to the degree of confidence with which we claim that an interaction really occurs in physiological conditions, since there is a hierarchy (albeit not absolute) of reliability of the various methodologies. This particularly useful piece of information can be easily incorporated in Zsyntax by labeling the Z-interaction to disambiguate which particular interaction we are referring to: 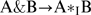
where I is an index running on the different classes of interaction. Of course, softwares for text mining and for predictions (see below) can be engineered to take into account the different classes of interaction only upon request.

#### 5. Subcellular localization

More or less in the same way, the issue of compartmentalization can be solved by Zsyntax. For, if we want to emphasize that a particular chain of reactions happens in a particular compartment, it is sufficient that we label suitably the derivability relation representing that process. That is, we could write:
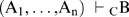
where C is an index running over the possible compartmentalization.

Similarly, by contextualizing the appropriate entities, or operators, or derivability relations, virtually every instance involving space constraints (recruitment to a particular subcellular localization, dynamic processes such as nucleo-cytoplasmic shuttling, progression through intracellular compartments such as during endocytosis and degradation) can be accounted for in Zsyntax, if the appropriate chain of molecular reactions is known.

#### 6. Quantitative aspects and the role of time

One aspect that might be apparently neglected by Zsyntax is the quantitative dimension of molecular interactions. This is especially true since Zsyntax is a logic, and one usually thinks of logic as a qualitative tool for formalization. However, as discussed above, Zsyntax is grounded into a particular kind of non-classical logic, well suited to retain and describe basic quantitative information. We have already explained how Zsyntax can account for reaction stoichiometries. In practice, this might be applied to many situation of interest for the molecular biologist. Let us imagine a situation in which two molecules A and B interact and we have also experimentally determined the stoichiometry of their interaction, be it 5%. The theorem could then be written as follows:
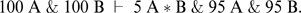



There are more complex ways, however, in which Zsyntax can incorporate quantitative aspects. For example, since it is a language specifically designed to take into account the strong empirical nature of molecular biology, and empirical information does frequently contain a temporal dimension embedded in it, we need to envision ways of incorporating a temporal dimension in Zsyntax-based theorems.

Before facing this aspect, we wish to mention the possibility of greatly enhancing the expressive power of Zsyntax by shifting from plain formulas, such as A, B, C, etc., to *labeled formulas*, such as A:α, B:β, C:γ, etc. (where α, β, γ are labeling strings, specifying the values of suitable parameters and ‘:’ is the sign indicating that the formula – e.g., A – is followed by its label – e.g., α). In this way, the labels allow us to specify any kind of additional information concerning the entities to which the formulas refer. Note that this manner of enriching the formal language is well-grounded in contemporary logic, where the so-called *Labeled Deductive Systems* is an active and innovative research area (see [32], [33])

Moreover the Z-interactions, which we have so far expressed by means of empirically valid formulas, could be more precisely written by means of *labeled rules*, telling us what is really happening. For instance, the elimination rule for the Z-conditional discussed above (→***E***: given A→B and A, then we can derive B; that is, the usual *modus ponens*), could be generalized as follows: 
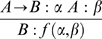
where α, β are strings of expressions denoting the values of suitable parameters, and f is a suitable function of the chosen parameters. Here, the purely logical mechanism of the *modus ponens* is integrated with a *labeling module* specifying the way in which the values of the parameters should be propagated by the application of the rule and, therefore, has an empirical content. The advantage of this approach is that the logical backbone of the process (expressed by the formulas) is separated from the additional empirical information that is required for a more accurate representation (expressed by the labels). In this way the basic structure of the process is maintained simple while providing a basis for increasingly accurate representations, depending on the amount of details that is captured by the labeling module.

Coming back to the temporal dimension, the Z-interaction that we have so far expressed by means of an empirically valid formula (A&B → A*B), could be written, for instance, in a more detailed and expressive way by means of the following *labeled rule*

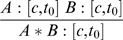
where, for example, c could represent the concentration, t_0_ the initial time of the reaction and t_∞_ a time sufficiently long to reach equilibrium. By the way, this rule would represent the Zsyntax equivalent of the equation of equilibrium binding, from which some kinetics parameters, including K_D_, could easily be derived.

Of course in this way we would only depict temporal snapshot, i.e. a static representation of the situation. On the other hand, if we wanted also the dynamics we should use differential equations. Although we have not explored this aspect, we suspect that this is exactly the point in which a logical language representing static situations of the system, such as Zsyntax, can be fused with a language (differential equations, be them ordinary – ODEs – or partial – PDEs) representing the dynamics of the system, in an attempt to allow also for the representation of the variations of the quantities involved.

The relationship between Zsyntax, ODEs and PDEs deserves further comments, as it could be argued that a Zsyntax reaction is a symbolic description that is very similar in structure to an ODE, or a PDE. However, there is a deep difference between the two approaches. To better illustrate such a difference, a brief digression into physics will be useful. Roughly speaking any physical theory has four formal levels: i) the level of the geometry in which the physical systems lie; ii) the level of the logic coding the allowed inferences among physical statements and therefore permitting the demonstrations of the theorems; iii) the level of the tools (sets of ordinary and partial differential equations – ODEs and PDEs) describing the dynamics of the involved systems; iv) the (meta)level of the language “enveloping” the first three levels. This four-layered structure can be grasped by looking at the Table 9.

**Table 9:** The four levels of mathematics into physics.

	“Enveloping” mathematics	Geometry	Logic	Dynamics (ODEs PDEs)
**Classical Mechanics**	Vectorial calculus	Euclidean geometry	Classic logic	Newton laws
**General Relativity**	Differential topology	Riemannian geometry	Classic logic	Einstein equations
**Quantum mechanics**	Complex functions and Hilbert spaces	Euclidean geometry	Classic logic plus Quantum logic	Schrödinger equation

As it should be evident, logic is the (more or less latent) inferential backbone of any physical theory, while the sets of ODEs or PDEs are the “muscles” permitting the representation of the evolution in time, in the ordinary 3-dimensional space, or in other spaces (as the phase space, or any other abstract n-dimensional space) of the systems.

What is the situation in molecular biology? Here, we do not have any “enveloping” mathematics (and it is unknown whether this will ever be possible); geometry needs not to be made explicit (as it is implicitly assumed that the space in which the molecules lie is Euclidean). Sometimes we use sets of ODEs or PDEs to model particular dynamics, as it happens, for example, in the case of gene regulatory networks [34]. But there is something more that should not be neglected, especially from a formal perspective: i.e. that molecular biologists do make inferences. Zsyntax is exactly a language that tries to capture and formalize this inferential backbone. Once fixed this backbone, analogously to what happens in the physical domain, the “attached” sets of ODEs or PDEs permit to represent the dynamics. Differently said, Zsyntax and ODEs/PDEs belong to two different “levels” (as from Table 9), those of Logic and of Dynamics, respectively. Needless to say, the way in which an a-temporal language (as Zsyntax is) and a temporal language (as usually ODEs and PDEs allow) could be linked has to be developed in depth. Here we simply envisage this possibility that, if achieved, should give us a more complete formal representation of what happens in the molecular biology domain.

This brings us to one final consideration concerning logic and ODEs or PDEs. Clearly, any time we integrate a set of ODEs or PDEs (that is, we solve it), there is an underlying logic that allows us to move from one step of the solution to another, and that the logic is the classical (Fregean) one. But, whenever we insert the group of statements concerning such a set of ODEs or PDEs and its integration into a scientific representation (be it a physical representation or a biological one permitted, for instance, by Zsyntax), the logic underlying the group has to be linked to the logic of the backbone of the representation. This is a very subtle question concerning the logical foundations of the scientific representations and its discussion would be beyond the scope of this paper. However, this is particularly evident in Quantum Mechanics where there are two different kinds of logic at work: the classic and the quantum one, which is non-classical. Classic logic is underlying any move from one statement to another, particularly when they belong to the demonstration of a theorem; but whenever the statements concern the non-distributive algebra of the quantum operators we have to set it aside and use quantum logic. The same would happen, more or less, with Zsyntax, which is based on a non-classical logic. Since we have claimed that Zsyntax might represent a logic backbone for the formal representation of molecular biology, this implies that, if also sets of ODEs and PDEs enter the representation, we would have to work with two different logics: the Fregean (classical) one underlying such sets and their integration, and the non-classical one allowing the backbone and the demonstrations of the theorems.

### Possible Applications of Zsyntax: Text Mining

The transposition of biological processes into a formal, logical language would allow text miners to use tools that have a number of advantages over the current ones available. There is overall agreement [21] that text mining involves: 1) information retrieval (finding the necessary information in available repositories); 2) named entity recognition (focusing in on relevant notions); 3) information extraction, i.e. drawing out the (relations among the) notions that we are looking for; 4) a question/answer task (having the possibility of asking for specific data, or data correlation, and the having the possibility of obtaining correct answers); 5) text synthesis (compacting the retrieved information). The use of logical tools for all these tasks has recently been advocated in the fast growing field of so-called description logics [35].

We can envision now what would happen if publications relating to biological processes had, not only the traditional abstract, but also the simplified version of the logical deduction, i.e., the theorems, representing these processes. The core information of any paper would then already be written in a way that could easily be handled by informaticians working in the field of information storage. This would lead relatively rapidly to the creation of a unique database containing information (written in a formal language) on biological reactions and on the molecules participating in these reactions, from which text miners could extract relevant information.

As an example let us consider again the complex series of interplays between MDM2 (both the gene and the protein) and TP53. Through the usage of classical Boolean operators in a PubMed search, one would retrieve more than 3,000 references for the query “p53 AND mdm2” (ignoring possible permutations with less used terms such as TP53 or hdm2) and more than 500 references for the query “p53 AND mdm2 AND interaction”. The derivations of even the simple theorems described herein would require many hours of work, sifting through largely irrelevant (for the purpose of the search) literature.

Of course many efforts are directed at the creation of more rationale, and user-friendly, databases, for example of biological interactions. These are based either on text mining algorithms (frequently based on natural language) that scan the literature (in general, titles an abstracts) and create bio-networks based on co-citation, or on manually-curated databases, or on a combination of the two. These databases are extremely useful, as they are also frequently linked to a large body of relevant information concerning taxonomy, biological function, publication, annotation, cross-reference, or even intellectual property. They address, however, a different need with respect to that would be addressed by a hypothetical Zsyntax-based database. As a case in point, we submitted the query “TP53, Mdm2” to two widely used databases, PubGene (www.pubgene.com) and BOND (bond.unleashedinformatics.com).

In PubGene the interaction between Mdm2 and p53 was promptly evidenced. By restricting the search to “based on co-occurrence with protein interaction keyword in the sentence”, we obtained 386 and 1219 entries (corresponding to papers), in *H. sapiens* and All Organisms, respectively. Entries were annotated with “interaction terms”, such as activates, interacts, downregulates, degrades and so on. This is a step forward with respect to a PubMed search, but would still require many hours of work to obtain the desired information.

In BOND, the search returned 107 “interactions”. The “interactions” (labeled with several useful qualifiers, such as experimental evidence, taxonomy, molecule labels and identifiers) contained many entries irrelevant for our quest (for instance of interactions of either TP53 or MDM2 with other molecules), but also some higher level of definition of relevant interactions. For instance, the complexes between TP53 and MDM2, and those between TP53 and the *MDM2* gene promoter were clearly distinguishable. We did not manage however to intuitively derive information on the regulation of the TP53:MDM2 complex by phosphorylation.

In a hypothetical Zsyntax-based database, on the other hand, the simple search with the keywords “TP53 & MDM2” (where & is our non-Boolean Z-conjunction) in the initial aggregate would return all of the theorems involving these two molecules in their exact molecular context. The integration of the Zsyntax language into high-resolution databases, such as BOND, would appear therefore as a decisive step forward.

### Possible Applications of Zsyntax: The Prediction of Biological Data and Reactions

We have shown how complex biological processes can be described in terms of logical deductions, which lead, from an initial aggregate (the premise) to a final aggregate (the conclusion of the deduction). Since, as previously mentioned, all the logical processes we have described are amenable to computer processing, it will be possible to automate the demonstration of theorems with the aid of suitable software programmed to work with Zsyntax. In its direct application, this means that it will be possible to develop an automated proof engine which takes as input an initial aggregate A_1_&…&A_n_ together with the conclusion B that we intend to reach, plus suitable heuristics. The output will be either the demonstration of the theorem or its rejection.

In case of rejection we can move backwards by reversing this process and find good indications on the reason why the search for a proof did not succeed. Implementing this reverse process means *thinking abductively* (hence, from the conclusion, we work backwards to re-construct the most likely pathway leading to this conclusion). This is what molecular biologists “intuitively” do routinely in the laboratory, and the method is extremely relevant to the scientific process, since backward reasoning: (a) is goal-oriented and (b) allows researchers to make predictions for new data and new reactions when deduction fails. Zsyntax can help goal-oriented theorem proving, in which researchers start from a conclusion, B, to look for the possible premises from which B can be derived. By applying the inference rules in the reverse order and by reiterating this inverse process, it will be possible to logically reconstruct several possible paths that lead to B. The initial node in each reconstructed path will be an aggregate from which the conclusion could be obtained, even though this might not match the experimental initial aggregate. Researchers can sift through all possible paths in the search tree (and using suitable heuristics can prune the wrong paths), to narrow down the choice until they finally arrive at a node that consists of the experimentally designed input aggregate. If the search fails, something is obviously missing from the logical reconstruction. However, even a failed search process usually contains enough information to provide a number of possible working hypotheses that can solve the initial problem, either by adjusting the premises, or by considering additional EVFs, something that automated theorem provers are able to do. In either case, the process may lead to new knowledge, because the relevance of the additional information may not have been previously perceived. To better grasp this point, let us consider an initial aggregate C  =  A_1_& …&A_n_. Up to now, we have applied our rules forward, starting from C and going towards the conclusion B, to show that C⊢ B. Suppose, however, that this is not true, that is, that C does not lead to B. This might happen because: (i) the initial aggregate C is not sufficient, and we must add new resources A_n+1_& …&A_n+k_, so that with C'  =  A_1_& …&A_n_& A_n+1_& …&A_n+k_, we have C' ⊢ B; (ii) there are additional EVFs that we have not taken into consideration. In the former case, finding the missing A_n+1_& …&A_n+k_ means finding new data; in the latter case, it means finding new reactions. These missing items can be found by using our logical rules backwards, that is, we could “abduce” (predict) them by taking advantage of the available empirical information (i.e., the EVFs) and of the logical rules. Ultimately, this leads to the generation of hypotheses that can be tested in the “wet” lab.

A simple example of how this can work in practice is the following one. We know the degradation path of TP53 from the literature [22]. In this pathway, a ubiquitin ligase, MDM2, interacts with TP53, leading to its ubiquitination, which in turn, destines ubiquitinated TP53 for proteasomal degradation [22], [29]. This pathway can be summarized in a theorem (where U stands for ubiquitin, and P for proteasome), whose thesis is the following: TP53&MDM2&U&P ⊢ d(TP53), as depicted in Table 10 in the simplified form of Zsyntax (note that this theorem represents a “sub-routine” of the general theorem of the regulatory loop of TP53 depicted in Table 4 and Table 5).

**Table 10:** Simplified version of the theorem concerning the degradation path of TP53.

**Theorem**
TP53&MDM2*&* U& P ⊢ d(TP53)
**Demonstration**
1. MDM2 & TP53 → MDM2*TP53
2. (MDM2 *TP53) & U → (MDM2*TP53) *U
3. (MDM2*TP53) *U → MDM2 & (TP53*U)
4. (TP53*U) & P → (TP53*U) *P
5. (TP53*U) *P → d(TP53) & U & P

We also know from the literature that another protein, NUMB, interacts with MDM2 [36]. This interaction can been described using an EVF: MDM2&NUMB→ MDM2*NUMB. Although MDM2 is a common denominator in this pathway and in that of TP53 degradation depicted above, until recently there were no connections between them. In the absence of experimental evidence, which only came in 2008 [37], but in the presence of an automated prover based on Zsyntax, would it have been possible to generate deductive paths (to be tested in the wet lab) linking an initial aggregate, TP53&MDM2&NUMB&U&P to the degradation of TP53? Assuming the existence of an appropriate database containing information about the path leading from TP53&MDM2&U&P to d(TP53), and about the reaction MDM2&NUMB → MDM2*NUMB, we could ask the automated prover to generate possible solutions to our problem. Given the initial aggregate, (TP53&MDM2&NUMB&U&P), there is a path from one of its subsets (TP53&MDM2&U&P), to d(TP53). This means that either 1) there is no role for the NUMB-MDM2 interaction, so no deductive path can be constructed in which all the items of the initial aggregate (TP53&MDM2&NUMB&U&P) are used, or that 2) NUMB might play a role in a situation of regulative loop. The solution of this problem requires a preliminary experimental step to assess whether there is a mechanistic connection between the levels of NUMB and those of TP53. This can be achieved, for instance, by modulating the levels of NUMB, by RNA interference and/or overexpression experiments, and checking the levels of TP53. We have demonstrated that is indeed the case [37], but these results do not per se suggest the molecular wiring of this putative loop. At this point the theorem-prover could check all possible interactions between the molecule MDM2*NUMB and the other molecules. The main problem lies in understanding which path should be coupled with the known MDM2/TP53/NUMB pathway in order to identify the correct regulatory loop. The theorem-prover can indicate all the logically possible paths, that is, the theorems representing hypothetical empirical paths. By means of suitable heuristics, the program prunes those that we already know to be biologically impossible (or unlikely). In this way, we are left with only a few hypotheses (see Table 11) that can be validated or disproved in the laboratory.

**Table 11:** The eight hypothetical theorems for the NUMB, TP53, MDM2 regulatory loop.

1	TP53&MDM2&NUMB&U&P ⊢ ((MDM2*NUMB) *TP53) &U&P
2	TP53&MDM2&NUMB&U&P ⊢ (MDM2*NUMB) &U& P& TP53
3	TP53&MDM2&NUMB&U&P ⊢ ((MDM2*NUMB) *U)&TP53&P
4	TP53&MDM2&NUMB&U&P ⊢ ((MDM2*NUMB) *P)&TP53&U
5	TP53&MDM2&NUMB&U&P ⊢ (MDM2*NUMB)&(TP53*U)& P
6	TP53&MDM2&NUMB&U&P ⊢ (MDM2*NUMB)&(TP53*P)& U
7	TP53&MDM2&NUMB&U&P ⊢ (MDM2*NUMB)&(U*P)&TP53
8	TP53&MDM2&NUMB&U&P ⊢ (MDM2*NUMB)&((TP53*P)*U)

What we have presented here is, obviously, an a posteriori case, in which the results of the final “wet” experiment are already known; indeed we have recently shown that the correct path is depicted by hypothesis 1 of the above Table 11 [37]. In this circuitry, the formation of a MDM2/NUMB/TP53 tricomplex inhibits the ubiquitin-ligase activity of MDM2, thereby preventing the ubiquitination of TP53 and its degradation. Of course, had the empirical test been negative, this would have meant that at least one item of data (a molecule), or possibly additional EVFs, were missing in the initial aggregate. In this case, the theorem prover might have provided some good indications on the type of molecule or EFV to look for. Although the simple example above is meant to exemplify the potential of Zsyntax as heuristic tool, it naturally does not illustrate its full computational power.

### Zsyntax and Other Languages

In the Introduction, we have already commented on the increasing efforts, especially in the field of bioinformatics, aimed at offering new languages and algorithms. In this section we would like to make some additional comments focused on specific projects. A comprehensive review is, of course, outside of the scope of this paper, but some points are worthy of mention.

Without any pretense of exhaustiveness, we can broadly identify two types of approaches: one aimed at the creation and the proposition of codes to standardize the collection, the storage and the retrieval of biological data, another concerned with the construction of biological networks to represent sets of inter-correlated data.

By the former we mean efforts such as the MIAME (Minimum Information About a Microarray Experiment) [38] or the MIAPE (Minimum Information About a Proteomics Experiment) [39], which are aimed the identification of standards to perform high-throughput experiments and to communicate the results. In the same category, we can include efforts to construct common platforms to exchange data on biological pathways (for example, BioPAX [40] see also www.biopax.org), and efforts, in the field of bio-ontology, to offer standards for biological information by HUPO (www.hupo.org) or HUGO (www.genenames.org).

Zsyntax does not belong to this category of biological rationalizations, even if, needless to say, on the one hand, it should use standard nomenclatures and, on the other hand, it could be considered as a coding tool for chains of biological reactions. This latter outcome does not represent, however, the objective of Zsyntax, but rather an obvious side product, since the adoption of any formal language implies standardization.

Different considerations apply to the case of network representations. In the last few years, increasing effort has been directed at the identification and representation of biological networks of protein-protein interaction, gene regulation, gene expression, metabolism, signal transduction, and so on. Notwithstanding the *prima facie* intuitiveness of the approach, it is becoming increasingly apparent that network representations suffer important semantic limitations. In particular, in many contexts the graph-based representations turn out to be rather unclear, and many iconic and pictorial representations (cartoons, arrows of different colors and shapes, added tables, etc.) [41] must be introduced to help the reader. Bioinformaticians are obviously conscious of this problem [42], [43] and actively working on possible solutions. In this framework, Kitano and coworkers have proposed a possible solution [44]. Their idea consists in realizing a codification of network representations that could be transferred into a machine-readable language apt for computational analysis. In particular, they proposed what is called the SBML (System Biology Markup Language), which should allow an unambiguous codification of the different situations that can be found in different kinds of biological networks. Such a language should also allow the exchange of information among different network representations (see www.sbml.org). The relevance of this approach lies in particular in the fact that it is already supported by a well-developed software that implements the proposed codification.

Zsyntax shares many features with this kind of approach, although there are substantial differences. The major one resides in the fact that network representation approaches are bottom-up. For, they start from already existing biological databases and try to represent them either pictorially or, in a more sophisticated way, by means of suitable software. Instead, Zsyntax proposes a top-down approach based on the concept of drawing out the logic implicit in molecular biology (both theoretically and experimentally) and, as an inevitable consequence, it allows also to deal with biological networks. However, Zsyntax is first and foremost a logic, that is a well-formulated (in a technical sense) language which is computable. Through Zsyntax, one can attain the same computational goals reached by bottom-up approaches, such as that of Kitano et al., by starting from a more theoretical point of view. The uniqueness of Zsyntax consists, however, in the ability to put together empirical information (in the EVFs) and formal rules (in the LVFs), in order to obtain a language that allows the representation of molecular biology reactions as theorems. This, in turn, permits to envision molecular biology as a collection of theorems, that is, as a branch of science writable in a deductive way (of course “deductive” should be taken in the sense explained above).

### Conclusions

We have shown that molecular biology processes can be thought of, and written as, logical deductions in the Zsyntax language, paving the way for their computational treatment. We foresee text mining as the prime field of application of Zsyntax. As Rzhetsky *et al*. [21] emphasize, “The current format of scientific journals follows a model established long before the era of computers, cheap electronic storage space, and digital publishing”. By circumventing the problems connected with the use of natural language, Zsyntax offers a “ready-to-use” formalization of biological reactions that may greatly aid text mining. In addition, the implementation of Zsyntax-based theorem provers may allow for the development of formal tools for biological prediction. This latter effort will obviously be challenging. On the on one hand, the development of Zsyntax-based dynamic tools can exploit the vast repertoire of knowledge that has been developed in the area of automated reasoning, and in particular in the fields of substructural logics and labeled deductive systems. On the other, the development of such a toolbox might run into problems common to other approaches, such as the *combinatorial explosion*, mentioned in the Introduction. Perhaps, similarly to what we discussed concerning the relationships between Zsyntax and ODEs/PDEs, this might represent another instance in which Zsyntax can be combined with other languages suitably developed to circumvent the problem [16], [17].
